# Mycobacterial Response to Organic Solvents and Possible Implications on Cross-Resistance With Antimicrobial Agents

**DOI:** 10.3389/fmicb.2018.00961

**Published:** 2018-05-15

**Authors:** Cátia Pacífico, Pedro Fernandes, Carla C. C. R. de Carvalho

**Affiliations:** ^1^Institute for Bioengineering and Biosciences, Department of Bioengineering, Instituto Superior Técnico, Universidade de Lisboa, Lisbon, Portugal; ^2^Faculty of Engineering, Universidade Lusófona, Lisbon, Portugal

**Keywords:** *Mycobacterium vaccae*, tolerance, cell adaptation, membrane, antibiotic, efflux pump inhibitor

## Abstract

*Mycobacterium vaccae*, a bacterium found in soil, has been receiving attention as adjuvant to antituberculosis treatment, vaccines and immunotherapies and even as antidepressant. This bacterium is also able to degrade several pollutants, including aromatic compounds. The increasing presence of organic solvents in the environment may lead to *M. vaccae* adapted populations. A possible relationship between solvent tolerance and decreased susceptibility to other types of chemicals, including antibiotics, may pose a problem during opportunistic infections. The present study thus aimed at assessing if solvent adapted cells presented higher tolerance to antibiotics and efflux pump inhibitors (EPIs). *M. vaccae* cells were able to thrive and grow in the presence of up 20% (v/v) glycerol, 5% (v/v) ethanol, 1% (v/v) methyl *tert*-butyl ether (MTBE) and 0.1% (v/v) toluene. During adaptation to increasing concentration of ethanol and MTBE, the cells changed their fatty acid profile, zeta potential and morphology. Adapted cells acquired an improved tolerance toward the EPIs thioridazine and omeprazole, but became more susceptible to the antibiotics levofloxacin and teicoplanin when compared with non-adapted cells.

## Introduction

Solvents are essential in several industries where they are used to dissolve solutes and as reaction media for low water soluble substrates and/or products. They are also widely used as degreasing and cleaning agents and in the formulation of paints, inks and adhesives, and sometimes end up reaching the environment. While some organic solvents such as hydrocarbons, aromatic compounds, alcohols and ketones are known pollutants, some of them have been considered as emerging pollutants. The latter include solvents used in pharmaceuticals, cosmetics and pesticides with effect on living organisms still unclear but with known endocrine disruption potential, and putative ecological and health effects.

Bacteria exposed to toxic organic solvents developed an array of mechanisms to thrive, including extrusion of solvents through efflux pumps (Isken and de Bont, 1996; Ramos et al., 1998; de Carvalho et al., 2014), enzymatic modification of the toxic compound (Tay et al., 1998; de Carvalho and da Fonseca, 2005; Tyagi et al., 2011), morphological changes (de Carvalho et al., 2004a), vesicle formation (Ramos et al., 2002; Baumgarten et al., 2012), accumulation of storage lipids (Cortes and de Carvalho, 2015), adjustments in the fatty acid composition of cells (Heipieper and de Bont, 1994; de Carvalho et al., 2005a; Nielsen et al., 2005; Unell et al., 2007; de Carvalho, 2012), modification of phospholipid headgroups (Ramos et al., 2002), phospholipid content and biosynthesis rate (Pinkart and White, 1997). These mechanisms have been extensively reviewed for Gram-negative bacteria but studies regarding tolerance mechanisms in Gram-positive bacteria have also been gaining interest.

Each bacterial strain possesses a certain level of tolerance toward organic solvents by genetic determination, although environmental factors may also play a role (Aono et al., 1994; Kobayashi et al., 1998). Some strains are even very efficient in degrading compounds that are regarded as toxic and lethal, such as toluene, by most bacterial cells (de Carvalho et al., 2007; Murínova and Dercová, 2014). The Corynebacteriaceae is the taxonomic family that harbors most Gram-positive bacteria with xenobiotic biodegradability capacity, including the highly efficient biodegraders *Gordonia, Rhodococcus* and *Mycobacterium* (Bell et al., 1988; Willumsen and Karlson, 1997; Arenskötter et al., 2004). *M. vaccae*, used in the present study, is known to metabolize, e.g., acetone, cyclohexane, styrene, benzene, ethylbenzene, propylbenzene, dioxane, and 1,2-dichloroethylene and to possess a co-oxidative capacity which results in the formation of intermediate molecules more amenable to mineralization than the initial recalcitrant compounds (Burback and Perry, 1993). This is an advantage regarding the inclusion of mycobacteria in bioremediation strategies or as biocatalysts in industrial processes. However, some nontuberculous mycobacteria are opportunistic pathogens and cause disease in humans and animals such as poultry and fish (Briancesco et al., 2013). Nevertheless, *M. vaccae* has been used as immunotherapeutic agent for tuberculosis treatment (Xu et al., 2009; Yang et al., 2011).

Since organic solvents are ubiquitous environmental contaminants and are present in disinfecting solutions for health care facilities and household cleaning products, it is important to assess if their contact with mycobacterial communities can favor the appearance of tolerant strains toward other chemicals such as anti-mycobacterial drugs. The solvents used in the present study were selected due to their known occurrence in the environment as pollutants. Methyl *tert*-butyl ether (MTBE) and ethanol are commonly used as additives of gasoline, since they can enhance its fuel octane value, which resulted in their widespread release into the environment (Deeb et al., 2003; Manzetti and Andersen, 2015). Contamination of groundwater with MTBE is often accompanied by aromatic compounds such as toluene (Wang and Deshusses, 2007). Aromatic and polyaromatic hydrocarbons are persistent organic pollutants mainly released to the environment by the petroleum industry and during storage and transportation of petroleum based products (Varjani et al., 2017). Lately, contamination with glycerol has been observed since this compound is the main byproduct formed during the production of biodiesel (Lemos et al., 2013). Both ethanol and glycerol are also recommended by the World Health Organization for handrub formulations and are commonly used in disinfectants and cleaning solutions (Penna et al., 2001; WHO, 2010). The aim of this study was thus the following: (i) to determine the tolerance of *M. vaccae* to ethanol, glycerol, MTBE and toluene and to assess the modifications occurring at the level of the fatty acid composition of the phospholipids of the cellular membrane during cell adaptation to the listed solvents; (ii) to adapt *M. vaccae* cells to the presence of organic solvents and assess if solvent-adapted cells present higher tolerance toward anti-mycobacterial drugs when compared with non-adapted cells.

## Materials and Methods

### Microorganism and Growth Conditions

*Mycobacterium vaccae* ATCC 15483 cells were grown in 100 mL Erlenmeyer flasks containing 20 mL of Mueller-Hinton (MH) broth supplemented with 0.1% Tween 80, in an Agitorb 200 incubator (Aralab) at 30°C and 200 rpm. Growth was monitored by optical density (OD) measurements at 600 nm.

#### Growth During Solvent Exposure

To assess the effect of organic solvents in the growth of *M. vaccae*, the tested solvent was added, at different concentrations, to mid-exponential phase cultures (optical density at 600 nm of 1.0 ± 0.2) growing in MH medium. Each assay was done at least in duplicate. Growth inhibition was determined in relation to non-stressed cells by calculating the ratio between the maximum growth rate attained in the presence of the solvent and that of the control cells grown in the absence of solvents. Growth was monitored and maintained under the same conditions as previously mentioned. Assays were done in duplicate.

#### Bacterial Adaptation to Solvents

A stepwise strategy was adopted to adapt bacteria as suggested previously (de Carvalho et al., 2007). Briefly, *M. vaccae* cells were grown in 100 mL Erlenmeyer flasks containing 40 mL of MH media supplemented with 0.1% Tween 80 and, once the culture reached mid-exponential phase, pulses of MTBE (to reach 1% v/v) or ethanol (to reach 5% v/v) were added. Further additions of solvent were made to the cultures whenever they reached mid-exponential phase. Growth was monitored and maintained under the same conditions as previously mentioned. Assays were done in duplicate.

### Chemicals

Mueller-Hinton broth was purchased from Sigma-Aldrich and Tween 80 from Merck-Schuchardt. The solvents used in this work were ethanol (>99.9%) from Panreac, toluene (>99.5%) from Riedel-de Häen, MTBE (>99.5%) from Fluka Analytical, and glycerol solution (86–89%) from Sigma-Aldrich. The antibiotics were levofloxacin and teicoplanin whilst the efflux pump inhibitors (EPIs) used were thioridazine and omeprazole, all from Sigma-Aldrich.

### Fatty Acid Composition

To evaluate the changes induced by each solvent, samples of 1 mL of cell suspension were collected before and during solvent exposure. Samples were centrifuged at 10,000 rpm during 5 min in a μSpeedFuge SFA13K from Savant Technologies, and the pellet was washed twice with mili-Q water. The cell fatty acids were simultaneously extracted from the cell pellet and methylated to fatty acid methyl esters (FAMEs) using the instant-FAME method from MIDI, Inc. The analysis were carried out in a gas chromatograph 6890N from Agilent Technologies, equipped with a flame ionization detector and an automatic injector 7683B, using a 25 m long Agilent J&W Ultra 2 capillary column. FAMEs were identified by the PLFAD1 method of Sherlock^®^ software version 6.2 from MIDI, Inc. The saturation degree was defined as the ratio between the sum of the percentage of saturated fatty acids and the sum of the percentage of monounsaturated fatty acids (MUFAs) present in the cells.

### Zeta Potential

Samples of 1 mL of cell suspension were collected before and during solvent exposure, washed three times with milli-Q water, and 40 μL were suspended in 2 mL of a 10 mM KNO_3_ solution. The electrophoretic mobility of mycobacterial cells was determined in a Doppler electrophoretic light scattering analyzer (Zetasizer Nano ZS, Malvern Instruments Ltd.) at 25°C, using a clear disposable zeta cell. The zeta potential was determined using the electrophoretic mobility as an indirect measure of cell surface charge, according to the method of Helmholtz-von Smoluchowski (Hiemenz and Rajagopalan, 1986). The zeta potential of the organic solvents was measured using a Glass “Dip” Cell, also from Malvern Instruments Ltd. Samples were prepared by adding 0.5 mL of solvent to 2 mL of milli-Q water. Measurements of water-miscible solvents were done by adding 40 μL of the solution to 2 mL of 10 mM KNO_3_. For water-immiscible solvents, where a second phase was formed, 1 mL of the aqueous phase was retrieved after centrifugation and added to 2 mL of mili-Q water. An aliquot of 40 μL of the mixture was collected and added to 2 mL of 10 mM KNO_3_. Calculations were performed using the Zetasizer software 7.10, from Malvern Instruments, Ltd. Assays were carried out at least in duplicate.

### Minimum Inhibitory Concentration Determination

The minimum inhibitory concentration (MIC) was determined for antibiotics and EPIs by the broth microdilution method in 96-well microtitre plates (Sarstedt Inc.) according to CLSI (2014). Antibiotics and EPIs were serially diluted in twofold steps, in 150 μL of MH broth, starting with the following initial concentrations: levofloxacin (10 and 7.5 μg/mL), teicoplanin (100 and 75 μg/mL), thioridazine (149.3 and 125 μg/mL) and omeprazole (500 and 400 μg/mL). To each well, 50 μL of cell suspension collected in the exponential phase and diluted in MH broth to reach a 0.5 McFarland standard was added. The plate was covered with a Breathe-Easy^TM^ sealing membrane (Sigma-Aldrich), and incubated at 30°C. OD was measured at 600 nm after ca. 72 h, at a wavelength of 600 nm, in a spectrophotometer SpectraMax^®^ 340 PC from Molecular Devices. The experiments were performed in duplicate, both for solvent-adapted and non-adapted cells.

### Fluorescence Microscopy

Cell viability and morphology were determined using a LIVE/DEAD^®^ *Bac*Light^TM^ Bacterial Viability Kit from Molecular Probes (Invitrogen, Thermo Fisher Scientific). The cells were observed using an Olympus CX40 microscope equipped with an Olympus U-RFL-T burner and a U-MWB mirror cube unit (excitation filter: BP450–480; barrier filter: BA515). Images were collected with an Evolution^TM^ MP 5.1 CCD color camera using the acquisition software Image-Pro Plus, both from Media Cybernetics. Cell viability was calculated by image analysis using Visilog 5 (Noesis SA) as previously described (de Carvalho et al., 2003).

## Results

### Growth of *M. vaccae* Cells Exposed to Organic Solvents

**FIGURE 1:**
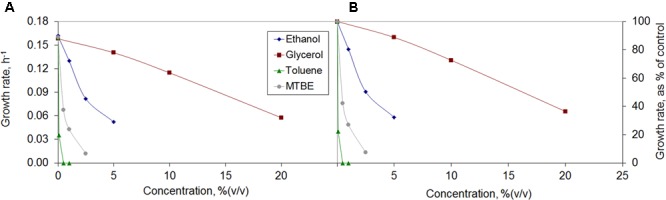
Growth rates of *Mycobacterium vaccae* cells attained **(A)** and growth inhibition observed relative to control **(B)** during exposure to the organic solvents ethanol, glycerol, toluene, and methyl *tert*-butyl ether (MTBE).

Some bacteria are tolerant to organic solvents, being able to grow in their presence. *M. vaccae* was able to grow in the presence of glycerol, ethanol, toluene, and MTBE, but the growth rate was highly affected (**Figure 1**). The growth rate decreased to a third of that observed in the absence of an organic solvent in the presence of 20% (v/v) glycerol, 3% ethanol, 1% MTBE and 0.1% toluene (**Figure 1B**). In all solvents, and particularly when water immiscible toluene and MTBE were used, a dose-dependent decrease in the growth rate was observed.

*Mycobacterium vaccae* cells were strongly inhibited by toluene and no growth was observed at 0.5% (v/v) toluene. This is in accordance with previous observations, since toluene concentrations as low as 0.1% (v/v) are usually sufficient to kill most microorganisms (de Carvalho et al., 2007). Nevertheless, the *M. vaccae* used in the present study was able to grow at toluene concentrations of 0.1%. *M. vaccae* ATCC 29678 (JOB-5) and *M. cosmeticum* byf-4 have been found to grow only in the presence of 0.01% toluene as sole carbon source (Burback and Perry, 1993; Zhang et al., 2013).

Although *M. vaccae* cells were strongly affected by the concentration of MTBE, visible by a sharp decrease in the growth rate with increasing MTBE concentrations, at 2.5% (v/v) the cells still grew at 7.4% of the growth rate of unchallenged cells (**Figure 1**). There are few reports of bacteria able to use MTBE as sole carbon source, including *Mycobacterium austroafricanum* IFP 2012 (François et al., 2002) and *M. austroafricanum* IFP 2015 (Ferreira et al., 2005). While the former was isolated from activated sludge of a waste water treatment plant (without a known contact with MTBE), the latter was isolated from the drain-water of a storage tank containing MTBE-supplemented gasoline. The *M. vaccae* ATCC 15483 used in the present study was isolated from dairy products from cow’s milk (Bonicke and Juhasz, 1964), where low amounts of MTBE are frequently found (Hiatt and Pia, 2004).

Higher concentrations of ethanol and glycerol than toluene or MTBE were tolerated by *M. vaccae* cells (**Figure 1**). These cells also grew must faster in the presence of ethanol and glycerol, reaching ca. 0.05 h^-1^ at 5% ethanol and 20% glycerol which is ca. one-third of the growth rate attained by unchallenged cells (**Figure 1**). Glycerol is generally used as carbon source to grow *Mycobacterium tuberculosis* and, since no glycerol uptake system is known, it has been considered that the rate of glycerol intake by passive diffusion may be sufficient for cell growth as the generation time for *M. tuberculosis* is 24 h (Niederweis, 2008). Although, in the present study, *M. vaccae* cells preferred the carbon sources present in Mueller Hinton broth and decreased the growth rate with increasing glycerol concentrations, glycerol is oxidized and assimilated by several *Mycobacterium* species, including *Mycobacterium smegmatis* and *Mycobacterium butyricum* (Hunter, 1953).

### Solvent Regulation of Membrane Fatty Acid Composition

**FIGURE 2:**
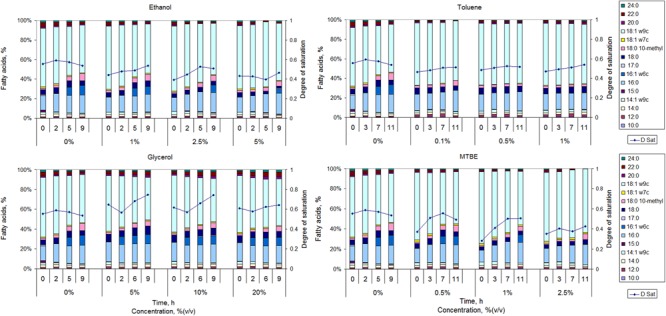
Fatty acid composition and respective calculated degree of saturation (D Sat) of *M. vaccae* cells grown in the absence of organic solvents and in the presence of ethanol, glycerol toluene, and MTBE.

The cells responded to the presence of organic solvents by making changes in the fatty acid content of the cellular membrane (**Figure 2**). The amount of solvent added decreased *M. vaccae* growth rate as mentioned in Section “Growth of *M. vaccae* cells exposed to organic solvents”, but at least 87% of the cells in the cultures represented in each data point of **Figure 1** were viable (data not shown), indicating that the large majority of the population adapted to the presence of the organic compounds at the concentrations tested.

When mid-exponentially growing cells were exposed to ethanol, the cells presented a lower degree of saturation of the fatty acids than unchallenged cells during the following 9 h. However, the degree of saturation increased with incubation time for ethanol exposed cells. This was mainly the result of an increase in the amount of 16:0 (<37% increase) and 17:0 (up to 3.5-fold) and a significant decrease in the amount of 14:1ω9c (decrease <52%), 18:0 (decrease <49%), 18:1ω9c (decrease <63%) and 20:0 (up to 100%). Cells exposed to toluene also presented a fatty acid profile with a lower degree of saturation than unchallenged cells at all tested time points (**Figure 2**). The degree of saturation of the fatty acids increased with time for all tested concentrations but the largest increase observed (for 1% toluene) was only ca. 14%. Cells exposed to toluene did not produce 16:1ω6c and maintained the content of 16:0 at ca. 17% and of 18:1ω9c at ca. 61%. However, the content of 18:0 10-methyl increased 3.8-, 2.6-, 1.1-, and 1.0-fold during the 11 h of exposure when the cells were exposed to 0, 0.1, 0.5, and 1%, respectively. A contrary effect was observed in *Rhodococcus opacus* strains where the content of 10-methyl branched fatty acids increased up to 34% when the cells were exposed to toluene, in comparison to cells grown in fructose (Tsitko et al., 1999). The largest increase was observed in the amount of 18:0 10-methyl, which occurred at the expense of unsaturated and not saturated fatty acids. Since the degree of saturation of toluene exposed cells only slightly increased, *M. vaccae* may prefer to decrease the content of branched fatty acids to decrease membrane fluidity.

An increase in the content of the branched fatty acid 18:0 10-methyl was observed with time in *M. vaccae* cells exposed to glycerol and MTBE, but the values attained were lower than the attained for non-exposed cells (**Figure 2**). Not only glycerol exposed cells presented a degree of saturation higher than non-exposed cells along time, but cells exposed to glycerol increased in general 2.9-fold the content of 18:0 10-methyl (as mentioned, a 3.8-fold increase was observed in unchallenged cells along time). MTBE exposed cells presented, in general, a lower degree of saturation than non-exposed cells along time, but its value increased 32–79% with exposure time, whilst a 4.4-, 3.7-, and 3.3-fold increase was observed in the amount of 18:0 10-methyl in cells exposed to 0.5, 1, and 2.5% MTBE, respectively. During exposure to both MTBE and glycerol, the cells responded by increasing the content of branched fatty acids with concomitant decrease in the content of MUFAs.

The synthesis of 16:1ω6c seems to be highly affected by the presence of organic solvents in *M. vaccae* (**Figure 2**). This fatty acid could be produced in cells exposed to all concentrations of ethanol and glycerol, reaching 7.6–9.2% of the lipid content of the cells. However, it could only be synthesized in cells exposed up to 1% MTBE and was not produced in the presence of toluene. The production of 16:1ω6c, which is relatively rare in nature, also decreased in *Rhodococcus* sp. 33 exposed to benzene (Gutiérrez et al., 1999) and increased in *Mycobacterium phlei* grown at 10°C when compared to those grown at 35°C (Suutari and Laakso, 1993), suggesting its contribution to the adequate fluidity of the cellular membrane.

### Cell Adaptation to High Concentrations of Solvents

To assess if *M. vaccae* cells adapted to organic solvents presented a higher tolerance to antibacterial drugs, cells were adapted to increasing concentrations of ethanol and MTBE throughout a period of approximately 70 h. These solvents were used due to their relevance in the environment as they are widely used, e.g., as gasoline additives. MTBE is capable of raising the octane number and is extensively used in free-lead gasoline. However, the water solubility of MTBE, its high mobility in the environment and low biodegradability, has led to a widespread contamination of groundwater and soil (Bonjar, 2005; Abbaspour et al., 2013). The encouragement for the use of ethanol as gasoline additive and as biofuel, made by several governments, also increased the number of ethanol-based fuel spills and environmental contamination with repercussions on microbial communities (Nelson et al., 2010; Leal et al., 2017). The ability of *Mycobacterium* strains to degrade such compounds and their presence in contaminated soil (Johnson et al., 2004, Johnsen et al., 2007; Ohkubo et al., 2009) justifies the assessment of the adaptation of *M. vaccae* to these compounds.

To adapt *M. vaccae* to both ethanol and MTBE, a fed-batch approach was used, as previously demonstrated for the adaptation of *Rhodococcus erythropolis* cells to terpenes and toluene (de Carvalho et al., 2005b, 2007). Since *M. vaccae* cells were able to thrive under concentrations of 5% (v/v) ethanol and 1% (v/v) MTBE, several pulses of solvent were added to the cultures whenever mid-exponential phase was reached. Briefly, the first pulse of solvent was applied after 16 h, when the cells were in the mid-exponential phase. Since more substrate was added, after an initial adaptation period, the cell cultures grew exponentially and another pulse of organic solvent was added. In total, three pulses of 5% (v/v) ethanol and four pulses of 1% (v/v) MTBE were added during the ca. 70 h.

#### Cell Morphology and Viability

**FIGURE 3:**
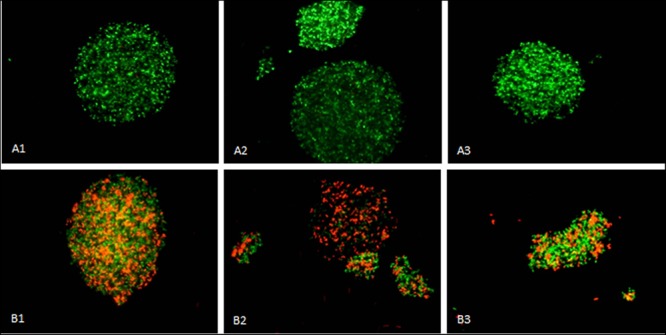
*Mycobacterium vaccae* cells grown in the presence of ethanol **(A1–A3)** and MTBE **(B1–B3)** presented extensive aggregation with the formation of clusters. Images 1–3 are from independent samples taken at 45 h of cultivation. Viable cells are stained green by Syto^®^9 whilst non-viable cells are stained red by propidium iodide. Horizontal field width: 800 μm; vertical field width: 600 μm.

After 45 h of growth under each condition, the cell viability and morphology was assessed using fluorescence microscopy. Viability of cells exposed to ethanol was higher (94.2%) than cells exposed to MTBE (48.8%). Cell aggregation was extensive in the presence of both solvents (**Figure 3**). Cell aggregation is influenced by the hydrophobicity as well as by the electrokinetic potential of the cell surface and substrate (Borrego et al., 2000). Cells in the presence of ethanol were 3.7% larger than those grown in the presence of MTBE, and presented a circularity value 12% higher. In the presence of MTBE, the cells were more elongated, but the difference in the area-to-volume ratio was only 2% if the cells are considered a prolate spheroid. However, cells exposed to ethanol and MTBE showed an area-to-volume ratio, respectively, 26.3 and 24.8% smaller than non-exposed cells. This suggests that the cells diminished their surface area to decrease exposure to the toxic environment.

#### Net Surface Charge

The surface charge of bacteria is associated to the composition of the cell envelope and the interactions of the cell with ions, particles and surfaces. This physicochemical property can affect the entry of metabolites and toxic compounds into the cells and bacteria-host interactions (Yeung and Grinstein, 2007; Raut et al., 2015; Malanovic and Lohner, 2016). Under physiological conditions, bacterial cells have negative surface charges but cells may change their charge in response to the growth conditions. *R. erythropolis* cells have been found to even become positive when grown in long-chain alkanes (de Carvalho et al., 2009).

**FIGURE 4:**
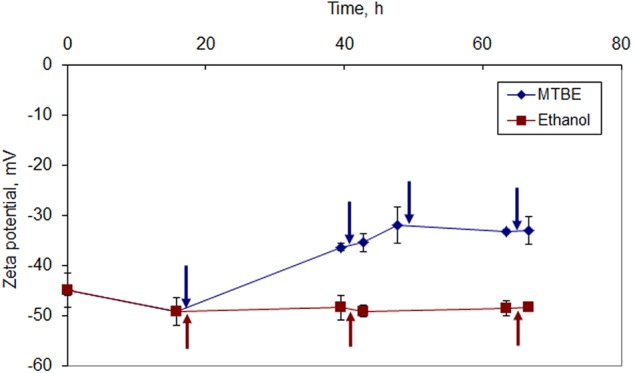
Zeta potential of *M. vaccae* cells during growth in the presence of ethanol and MTBE. The first addition of solvent was done to reach an initial concentration of ethanol of 1% (v/v) and 5% (v/v) MTBE. The same respective amount was added at the times indicated by arrows.

*Mycobacterium vaccae* cells grown on MTBE presented a less negative character than cells grown on ethanol at all data points (**Figure 4**). After the third ethanol addition, the cells presented a zeta potential of -48.4 ± 0.2 mV, which is only 1.9% higher than the net surface charge presented by cells before exposure to the solvent. On the other hand, after the third and fourth MTBE additions, *M. vaccae* cells presented an almost constant zeta potential around -33 mV, which is 44.6% less negative than that presented by cells prior to MTBE exposure.

Since the zeta potential is a measure of the electrostatic attraction/repulsion between particles, by decreasing the zeta potential values, cells growing in the presence of MTBE decreased the repulsion between themselves. This should have favored the observed cell aggregation. Cells grown in the presence of ethanol, where cell clustering was also considerable, apparently did not required adjustments in the net surface charge values.

#### Fatty Acid Composition

**FIGURE 5:**
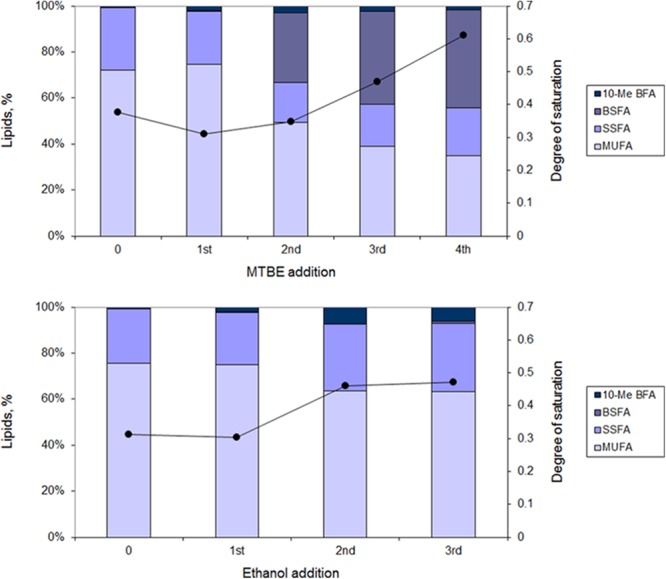
Lipid composition (bars) and degree of saturation (line) of fatty acids of *M. vaccae* cells during adaptation to MTBE **(Upper)** and ethanol **(Lower)**. The lipid composition was measured 1 h following addition of the organic solvent. MUFAs, monounsaturated fatty acids; SSFAs, saturated straight fatty acids; BSFAs, branched saturated fatty acids; 10-Me BFA, 10-methyl branched fatty acids.

During growth in increasing organic solvent concentrations, *M. vaccae* cells adapted their lipid composition. However, cells presented a fatty acid composition dependent on the solvent (**Figure 5**). Cells grown in the presence of MTBE, increased the amount of branched saturated fatty acids (BSFAs) with MTBE additions from 0.4% after the 1st to 42.0% after the 4th addition. This occurred with concomitant decrease in the amount of MUFAs from 73.9 to 34.5%. The main fatty acids involved were 15:0 *anteiso*, 15:0 *iso*, 17:0 *anteiso*, 17:0 *iso*, and 18:1ω9c. Since the amount of saturated straight fatty acids (SSFAs) was maintained between 22.9–20.9%, the degree of saturation of the cells during MTBE adaptation nearly doubled at the expenses of the decrease observed in the content of MUFA.

During adaptation of *M. vaccae* cells to ethanol, the production of BSFA was, however, very limited, reaching only 0.8% of the lipid content after the 3rd ethanol addition (**Figure 5**). In this case, the most important changes observed after the 3rd ethanol addition were a 17.1% decrease in the content of MUFA and an increase of 24.5% in the amount of SSFA along time. An increase in the content of 10-methyl branched fatty acids (10-me BFA) up to 6% of the total lipids was also attained after the final pulse of ethanol.

### Tolerance of Solvent Adapted Cells to Antibiotics and Efflux Pump Inhibitors

The MIC for the antibiotics teicoplanin and levofloxacin, and for the EPIs thioridazine and omeprazole, were determined for both non-adapted and solvent-adapted cells by the broth microdilution method. Solvent-adapted cells were shown to be more susceptible to teicoplanin than non-adapted cells: MTBE-adapted cells endured half the concentration whilst ethanol-adapted cells only grew in less than 25% of the concentration tolerated by non-adapted cells (**Table 1**). The MICs obtained for levofloxacin showed that both MTBE-adapted and non-adapted cells could grow up to a concentration of 0.6 μg/mL, whilst ethanol-adapted cells could only thrive in the presence of 0.04 μg/mL of levofloxacin. *M. vaccae* cells adapted to both solvents presented a fourfold increase in the thioridazine MIC value and a twofold increase in the MIC of omeprazole, when compared to the MIC of non-adapted cells.

**Table 1:** Minimum inhibitory concentration (MIC) of the antibiotics teicoplanin and levofloxacin and of the efflux pump inhibitors (EPIs) thioridazine and omeprazole toward *M. vaccae* cells not adapted to organic solvents and adapted to MTBE and ethanol.

	MIC (μg/mL)				
Non-adapted cells	MTBE-adapted cells	Ethanol-adapted cells
Teicoplanin	>100	50	25
Levofloxacin	0.6	0.6	0.04
Thioridazine	18.7	74.6	74.6
Omeprazole	250	500	500

**FIGURE 6:**
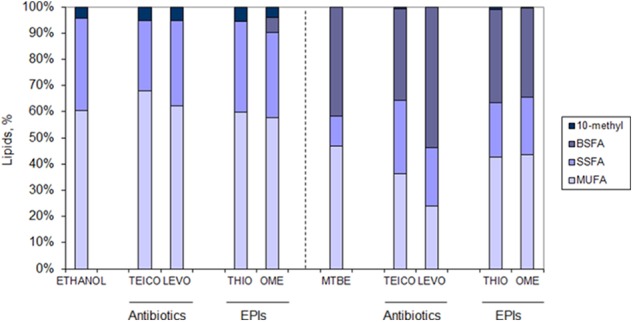
Lipid composition of *M. vaccae* cells adapted to ethanol (Left) and MTBE (Right) and exposed to ½ MIC of the antibiotics teicoplanin (TEICO) and levofloxacin (LEVO) and to ½ MIC of the efflux pump inhibitors (EPIs) thioridazine (THIO) and omeprazole (OME) after 41 h of cell growth (mid-exponential phase). Fatty acid class abbreviation as in **Figure 5**.

Cells adapted to ethanol and MTBE were grown in the presence of ½ MIC of antibiotics/EPIs and solvent to further assess the changes occurring at the lipid level in the presence of multistresses. While cells adapted to ethanol increased the content of MUFA when grown in the presence of ½ MIC of teicoplanin, cells grown in the presence of levofloxacin presented a lipid composition similar to cells adapted to ethanol but not exposed to antibiotics (**Figure 6**). The same was observed when the same cells were grown in the presence of ½ MIC of the EPI thioridazine. However, ethanol adapted cells grown with ½ MIC of omeprazole decreased the amount of SSFA with concomitant production of BSFA. These fatty acids were produced in high amount in MTBE adapted cells, as previously mentioned, and their content was further increased 30% in cells exposed to ½ MIC of levofloxacin (**Figure 6**). On the other had, the amount of BSFA decreased on average 19.6% on MTBE adapted cells exposed to teicoplanin, thioridazine and omeprazole, while increasing 30.6% in cells exposed to levofloxacin. A general decrease was observed in the content of MUFA in cells exposed to both antibiotics and efflux pumps inhibitors, when compared to those not exposed, which was accompanied by a twofold increase in the amount of SSFA.

Regarding the degree of saturation of the fatty acids of the cells, those adapted to ethanol only changed this parameter in the presence of teicoplain: the degree of saturation decreased from 0.6 in non-exposed cells to 0.4 in cells exposed to the antibiotic (data not shown). However, *M. vaccae* cells adapted to MTBE changed the degree of saturation of their fatty acids considerably: cells exposed to the EPIs increased this parameter twofold whilst cells exposed to the antibiotics increased the degree of saturation at least threefold (data not shown). The MTBE-exposed cells thus required a decrease in the fluidity of the membrane to cope with the extra challenge.

## Discussion

The environmental *M. vaccae*, which is considered to grow rapidly in comparison to other species of the genus *Mycobacterium*, has been receiving important attention as several studies have shown important exposure related health benefits. As probiotic, ingestion of *M. vaccae* was found to decrease anxiety and improve learning in mice (Matthews and Jenks, 2013). Recent tests in rats immunized with heat killed *M. vaccae* preparation also suggest the potential of immunoregulatory strategies to prevent stress- and trauma-related psychiatric disorders (Fox et al., 2017). It has been found that heat-killed *M. vaccae* may be useful as adjunctive to antituberculosis chemotherapy by clinical trials in newly diagnosed pulmonary tuberculosis human patients (Johnson et al., 2000), and as adjuvant for vaccines and immunotherapies (Skinner et al., 2001; Huang and Hsieh, 2017). Besides, a randomized phase III study showed that killed *M. vaccae* cells significantly improved the quality of live, by increasing cognitive functioning and vitality, of advanced lung cancer patients without affecting their overall survival time (O’Brien et al., 2004).

Although regarded as non-pathogen, *M. vaccae* has been associated to pulmonary and skin infections (Hachem et al., 1996; Katoch, 2004). *M. vaccae* may be found in the environment, and human contact with this species may be facilitated during outdoor activities and through agricultural products, but also by water distribution systems (Falkinham, 2009). The remarkable solute-stress tolerance of mycobacteria has been linked to their environmental tenacity (Santos et al., 2015). However, the major contribution for the ecology and epidemiology of nontuberculous mycobacteria is the effectiveness of hydrophobic cell envelope, which acts as a permeability barrier, containing mycolic acids arranged predominantly in a direction perpendicular to the cell wall surface (Nikaido et al., 1993; Hoffmann et al., 2008). Although the exact composition of the mycobacterial cell envelop is still being studied, most recent models propose an outermost layer, a cell wall and a conventional plasma membrane (Daffé et al., 2017; Rodriguez-Rivera et al., 2017). The latter is mainly composed by phospholipids such as cardiolipin, phosphatidylethanolamine, phosphatidylinositol, and glycosylated phosphatidylinositols (Crellin et al., 2013; Chiaradia et al., 2017). The particular lipid composition of mycobacteria has been related to resistance to disinfectants and antibiotics.

It has been suggested that environmental conditions may influence bacterial susceptibility to antimicrobials by promoting stress responses that result in altered gene expression patterns and cell physiology (Fernandes et al., 2003; Poole, 2012). The aim of the present study was thus to assess if cells exposed and adapted to the presence of organic solvents presented higher tolerance to antimicrobial agents than those non-adapted, and the role of lipids to this adaptation.

The growth rate of *M. vaccae* ATCC 15483 decreased to a third of that observed in the absence of an organic solvent in the presence of 20% (v/v) glycerol, 3% ethanol, 1% MTBE, and 0.1% toluene. The most hydrophobic solvents tested thus caused highest bacterial growth inhibition. MTBE is a widely used gasoline additive often found in contaminated soil and water due to fuel leakage from storage tanks and during transportation. Toluene can enter cellular membranes and in Gram-negative bacteria it has been shown that it causes a transition from a lamellar bilayer state to a hexagonal state, resulting in removal of lipids and proteins from the membrane and changes in membrane potential (Heipieper et al., 1994; Sikkema et al., 1995). In the Gram-positive *Staphylococcus haemolyticus*, no significant changes in phospholipids abundance or distribution was observed in cells grown with and without toluene, but the cells changed significantly their fatty acid profile (Nielsen et al., 2005). Curiously, *S. haemolyticus* increased membrane fluidity when exposed to toluene, contrarily to common gram-negative bacteria which make the cytoplasmic membrane less fluid. *R. erythropolis* DCL14 also increased the amount of branched fatty acids while decreasing in 40% the content of straight-chain fatty acids (de Carvalho et al., 2007). In the case of *M. vaccae*, the cells increased the degree of saturation of their fatty acids with exposure time to all organic solvents tested, but while the value was lower than that presented by non-exposed cells when they were exposed to ethanol, MTBE and toluene, cells exposed to glycerol presented a higher degree of saturation than non-exposed cells. Jarlier and Nikaido (1990) calculated the permeability of *M. chelonei* to glycerol and found that the *K*_M_ of the overall transport of glycerol to be 200 μM, suggesting a low permeability of the cell envelop to this small molecule. The low concentration of glycerol thus permitted in the interior of the cells may explain the high concentration of glycerol tolerated by *M. vaccae* cells in the environment.

When *M. vaccae* cells were allowed to adapt to increasing concentrations of ethanol and MTBE, by adding solvent pulses during cultivation, the cells apparently responded by decreasing membrane fluidity as result of a significant decrease in the amount of MUFAs. This resulted in an increase in the degree of saturation of the fatty acids with the amount of solvent added. However, the cells exposed to ethanol produced larger amounts of 10-methyl 18:0 (up to 6.1% of total lipids), whilst MTBE adapted cells increased the content of methyl branched fatty acids up to 42% of total lipids, particularly those *iso* branched. Poger et al. (2014) showed that fatty acid branching led to increased membrane fluidity since branches increased the area per lipid, reduced the bilayer thickness, lowered chain ordering, and led to the formation of kinks at the branching point. The results of the present study suggest that, during adaptation to both ethanol and MTBE, *M. vaccae* cells thus produced the fatty acids required to maintain the fluidity level necessary for cell survival and growth.

The alterations occurring at the lipid level were accompanied by changes in the zeta potential of *M. vaccae* cells which considerably decreased the negative character of their surface during adaptation to increasing concentrations of MTBE. This could explain the extensive cell aggregation observed in adapted *M. vaccae* cultures. However, cells adapted to ethanol, where aggregation was also extensive, did not change their zeta potential during adaptation. The aggregation was also favored by an area-to-volume ratio of solvent exposed cells smaller than that observed for non-exposed cells. Formation of large clusters of cells had been also observed during *Mycobacterium* sp. NRRL B-3805 exposure to both miscible and immiscible organic solvents, allowing protection of part of the cells from direct exposure to toxic molecules (de Carvalho et al., 2004b).

Cells adapted to organic solvents presented lower tolerance to teicoplanin and levofloxacin than non-adapted cells, although MTBE-adapted cells could endure higher concentration of both antibiotics than ethanol adapted cells. This could be the result of the changes in the fatty acid profile that MTBE-adapted cells carried out in the presence of the antibiotics, which resulted in a threefold increase of the degree of saturation. The glycopeptide teicoplanin affects peptidoglycan biosynthesis by binding to the D-Ala-D-Ala terminus of the pentapeptide side chains of peptidoglycan precursors, thus preventing polymerization reactions (Parenti, 1986). Teicoplanin is able to cross membranes due to the hydrophobicity of the molecule since the acyl substitute of the *N-*acylglucosamine is a fatty acid containing 10–11 carbon atoms (Parenti, 1986), which apparently anchors the antibiotic to the bacterial membrane (Westwell et al., 1995). This was considered the reason why teicoplanin induced more changes in the lipid profile of *Staphylococcus aureus* cells than vancomycin (Gonçalves and de Carvalho, 2016). Keiser et al. showed the organization of peptidoglycan synthesis into networks with varying drug susceptibility in *M. tuberculosis* (Kieser et al., 2015). The mutants produced presented different susceptibility to drugs affecting the synthesis of cell-wall components but not to the detergent sodium dodecyl sulfate. This suggested that cell-wall synthesis and not altered bacterial permeability was responsible for the altered drug susceptibility. This is in accordance to the results observed in the present study.

Levofloxacin which is emerging as promising antibiotic against rapidly growing mycobacteria (Pang et al., 2015), is a fluoroquinolone antibiotic presenting a zwitterionic and an uncharged neutral form but both cross membranes via passive transport (Cramariuc et al., 2012). The neutral form easily permeates through a lipid bilayer, whilst the zwitterionic molecules form stacks with reduced polarity which favor their entrance into the bilayer (Cramariuc et al., 2012). Fluoroquinolones are becoming selected drugs for the treatment of multi-drug resistant tuberculosis, in particular levofloxacin due to its broad spectrum and activity against *M. tuberculosis* (Alvarez et al., 2014). However, when Sarathy et al. (2013) analyzed the accumulation of fluoroquinolones in nutrient-starved *M. tuberculosis*, they observed a marked loss of bactericidal activity in starved cells in comparison with growing cells. One of the reasons pointed out by the authors was the altered cellular permeability.

*Mycobacterium vaccae* cells adapted to both MTBE and ethanol also adjusted their fatty acid profile in the presence of the EPIs thioridazine and omeprazole. These compounds help to increase the activity of antibiotics by inhibiting the efflux pumps responsible for their removal, which results in a decrease of the MIC of the antibiotic when the inhibitor is present (Coelho et al., 2015). Thioridazine has been presented as an effective therapy for the treatment of pulmonary tuberculosis regardless of the antibiotic resistance phenotype of the *M. tuberculosis* strain (Amaral and Viveiros, 2017). Thioridazine was also suggested for the treatment of *Mycobacterium avium* complex (MAC) infections. The number of chronic infections caused by MAC are surpassing tuberculosis in the United States (Deshpande et al., 2016). The proton pump inhibitor omeprazole is effective in several eukaryotic and prokaryotic cells (da Silva et al., 2011) and it also presents antibacterial effects against several bacterial strains including *Helicobacter pylori* (Jonkers et al., 1996). However, it has been suggested that omeprazole may promote the growth of *M. tuberculosis* and MAC bacteria due to a high prevalence of infections caused by these bacteria in patients using acid-suppressive agents (Suzuki et al., 2000; Hsu et al., 2014).

It has been shown that long-term exposure of *M. tuberculosis* to thioridazine increased the cell-envelope permeability (de Keijzer et al., 2016). However, MTBE adapted *M. vaccae* cells were able, apparently, to counteract this effect since a twofold increase in the degree of saturation was observed. Although the changes observed in ethanol adapted cells during growth in ½ MIC of both EPIs were not as remarkable as those observed in MTBE adapted cells, in both cases adapted cells presented significantly higher MIC values in comparison to non-adapted cells. Since efflux pumps should be able to pump antibiotics and organic solvents (Fernandes et al., 2003; de Carvalho et al., 2014; Blanco et al., 2016), our results suggest that adaptation to MTBE and ethanol not only involved adaptations at the lipid level but also the number and/or activity of the efflux pumps. Further studies involving different classes of solvents, antibiotics and efflux pumps are required for the fully understanding of how bacterial exposure to disinfectants and pollutants might influence the efficacy of antibacterial compounds. The recent attention that *M. vaccae* has been receiving as adjuvant for tuberculosis treatment, vaccines and immunotherapies makes this study relevant. Nevertheless, it should be also interesting to assess if exposure of *M. tuberculosis* to solvents also influences antituberculosis treatment.

## Author Contributions

CdC designed the experiments and wrote the manuscript. CP performed the experiments. CP and CdC analyzed the data. PF discussed the results and reviewed the manuscript.

## Conflict of Interest Statement

The authors declare that the research was conducted in the absence of any commercial or financial relationships that could be construed as a potential conflict of interest.
